# Impact of Concomitant Thoracic Trauma on Functional Outcomes After Surgical Treatment of Glenoid Fractures

**DOI:** 10.3390/jcm15093378

**Published:** 2026-04-28

**Authors:** Haluk Yaka, Muzaffer Harmankaya, Hasan Rüzgar, Ali Adem, İnci Hazal Ayas, Mustafa Özer, Ulunay Kanatlı

**Affiliations:** 1 Orthopaedics and Traumatology, Necmettin Erbakan University, Konya 42090, Turkeyaliadem51@gmail.com (A.A.);; 2 Department of Physiotherapy and Rehabilitation, Gazi University, Ankara 06490, Turkey; 3 Orthopaedics and Traumatology, Gazi University, Ankara 06560, Turkey

**Keywords:** glenoid fracture, posterior deltoid-sparing approach, Ideberg classification, mini-plate, minimally invasive surgery, scapula fracture, functional outcomes, thoracic trauma

## Abstract

**Background/Objectives:** The minimally invasive posterior deltoid-sparing (MIPDS) approach has been described for glenoid fractures; however, its outcomes for Ideberg type Ib, II, III, IV, and V fractures and the influence of concomitant injuries on functional recovery remain poorly understood. This study aimed to report minimum 2-year functional outcomes of these fracture types treated with the MIPDS approach using mini-plates, and to investigate the effect of concomitant thoracic trauma on clinical outcomes. **Methods:** Thirty-one patients with operatively treated glenoid fossa fractures were stratified into three groups: isolated glenoid fracture, concomitant thoracic trauma, and concomitant ipsilateral upper extremity fracture. Functional outcomes were assessed using the Constant, UCLA, and DASH scores at a minimum follow-up of 2 years. **Results:** No postoperative infection or nonunion occurred. Mean union time was 9.4 ± 2.4 weeks. Patients with thoracic trauma demonstrated significantly worse functional outcomes across all three scores compared to both other groups: lower Constant scores (70.9 ± 7.5 vs. 85.5 ± 5.9 and 82.6 ± 11.7; *p* = 0.012 and *p* = 0.042), lower UCLA scores (24.6 ± 7.9 vs. 32.5 ± 3.0 and 31.1 ± 3.2; *p* = 0.010 and *p* = 0.012), and higher DASH scores (29.3 ± 14.2 vs. 7.9 ± 9.2 and 9.5 ± 9.9; *p* = 0.003 and *p* = 0.006). Multivariate linear regression confirmed thoracic trauma as an independent predictor of higher DASH scores (β = 12.75, 95% CI: 2.00–23.50, *p* = 0.031, R^2^ = 0.344). **Conclusions:** The MIPDS approach provides safe and effective fixation for Ideberg type Ib, II, III, IV, and V glenoid fractures with satisfactory functional outcomes at minimum 2-year follow-up. Concomitant thoracic trauma is a significant negative predictor of functional recovery, and the possibility of inferior functional outcomes in this patient group should be considered.

## 1. Introduction

Scapular fractures account for less than 1% of all fractures in the adult population and approximately 3–5% of shoulder girdle injuries [1,2]. The most common type of scapular fracture is scapular body fracture (52%), and the second most common type is glenoid fossa fracture [3]. Historically, most scapular fractures have been managed conservatively, and several studies have reported satisfactory results with non-operative treatment [4,5]. However, reliable conclusions from the literature are difficult to draw, as studies investigating conservative treatment rarely specify the degree of fracture displacement [4]. Given that displaced scapular fractures can lead to instability, chronic pain, glenohumeral instability, and persistent symptoms, there has been an increasing trend toward surgical management [6,7].

The Judet approach has been widely used in prior studies with good reported outcomes; however, it is associated with significant damage to the deltoid muscle [8,9]. In 2011, Gauger and Cole described a minimally invasive technique for scapular neck and body fractures, in which the posterior deltoid is retracted superiorly through a lateral wall incision, thereby preserving the muscle [10]. In 2023, van de Wall et al. reported the surgical outcomes of this technique in 35 patients with scapular body, neck, and simple intra-articular fractures [11]. Subsequently, Lim et al. described the posterior deltoid-sparing approach specifically for displaced inferior and posterior glenoid fossa fractures [8].

The aim of this study was to report that Ideberg type Ib, II, III, IV, and V glenoid fractures can be treated surgically with the MIPDS approach using mini-plates with satisfactory clinical results for at least 2 years, and to investigate the influence of concomitant injuries—particularly thoracic trauma—on functional outcomes.

## 2. Materials and Methods

**Figure 1:**
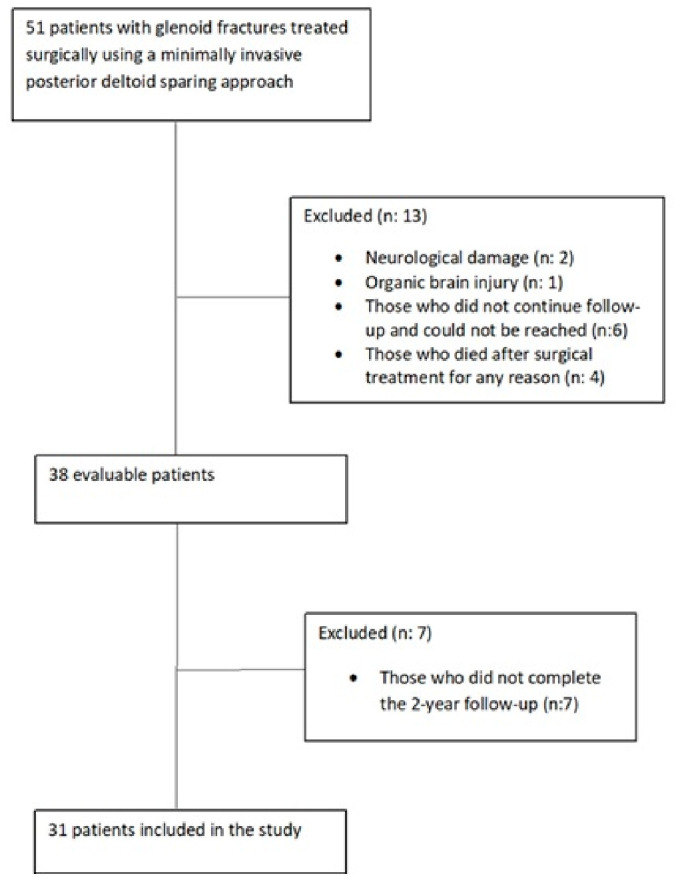
Participant flowchart showing inclusion and exclusion criteria.

Patients who underwent surgical treatment with the MIPDS approach for glenoid fossa fractures between February 2015 and January 2022 were retrospectively analyzed. Of 51 patients who underwent surgery, 31 with at least 24 months of follow-up were included. Patients with neurological damage such as brachial plexus injury (*n* = 2) and organic brain injury (*n* = 1), those who did not continue follow-up and could not be reached (*n* = 6), those who died after surgery for any reason (*n* = 4), and those who did not complete the 2-year follow-up (*n* = 7) were excluded (Figure 1). The mean age of the 31 included patients was 46.3 ± 16.6 years (range: 19–75), with 24 male and 7 female patients (Table 1). All patients met the previously defined surgical indication criteria (a gap or step-off of ≥4 mm) [12].

All patients sustained high-energy trauma resulting from traffic accidents, falls from heights, or work-related accidents. Eight patients had isolated glenoid fractures. Fourteen patients had concomitant thoracic trauma (lung contusion, rib fractures, pneumothorax, hemothorax), and 9 patients had additional fractures of the ipsilateral upper extremity (Table 2). Three patients had both concomitant thoracic trauma and ipsilateral upper extremity fractures; these patients were excluded from comparative group analysis as the statistical software identified constant values within this subgroup due to the small sample size. According to the Ideberg classification, there were 9 type Ib, 10 type II, 2 type III, 6 type IV, and 4 type V fractures (Table 3).

### 2.1. Surgical Technique

**Figure 2:**
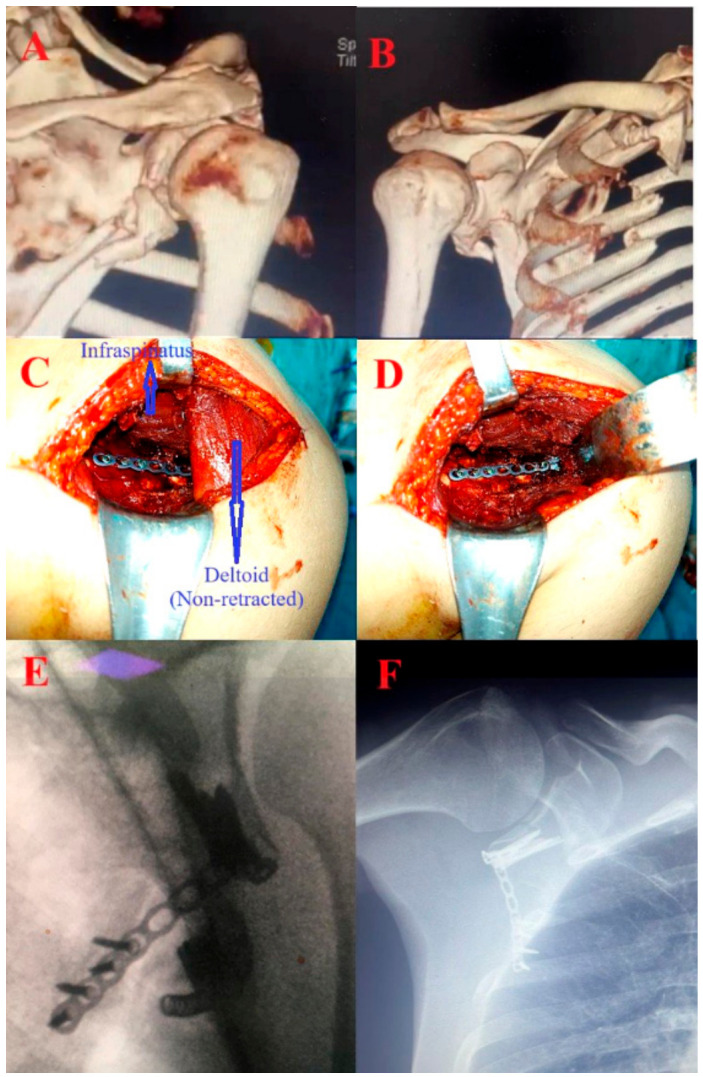
Representative intraoperative and radiographic images. (**A**) Preoperative three-dimensional computed tomography, posterior view. (**B**) Preoperative three-dimensional computed tomography, anterior view. (**C**) Intraoperative view after open reduction and internal fixation (ORIF) with the deltoid muscle in its native, non-retracted position. The infraspinatus muscle (upper arrow) and deltoid muscle (lower arrow) are indicated. The teres minor muscle lies immediately deep to the inferior retractor and is not visible in this view. (**D**) Intraoperative view after ORIF with the deltoid retracted superiorly by a retractor, providing wider exposure of the posterior glenoid and the underlying musculature. (**E**) Intraoperative fluoroscopic image confirming implant position after ORIF. (**F**) Postoperative anteroposterior radiograph demonstrating implant position and fracture reduction.

All patients were managed according to the principles of multi-trauma and damage-controlled surgery. With the patient in the lateral decubitus position, a longitudinal incision was made at the level of the lateral wall of the scapula, and dissection was carried out medially and laterally to the deltoid muscle. The inferior border of the deltoid was visualized (Figure 2A). The deltoid was retracted superiorly, and the infraspinatus and teres minor muscles were identified beneath. Blunt dissection was performed between these muscles; the infraspinatus was retracted proximally and the teres minor distally, exposing the lateral aspect of the infraspinous fossa and the posterior joint capsule. Open reduction was achieved and fixed with 2.0 mm or 2.7 mm L- or T-shaped locking plates, with headless compression screws (Xrbest^®^, Xrbest Medical, Zhangjiagang, Suzhou, China) used when necessary for articular surface restoration (Figure 2C,D). In fractures requiring additional fixation at the scapular spine or medial wall, supplementary incisions and implants were used. The posterior joint capsule was not opened unless necessary. All cases were finalized under fluoroscopic guidance (Figure 2E,F). All surgical procedures were performed by the same surgical team. Follow-up examinations were conducted at 3 weeks, 6 weeks, 6 months, and annually thereafter. Final data collection was performed in December 2024. Postoperative rehabilitation was tailored to each patient’s overall injury burden and general condition, particularly in cases of concomitant thoracic trauma or ipsilateral upper extremity fractures. In general, pendulum exercises were initiated as pain allowed in the early postoperative period, with progressive active range-of-motion exercises commenced following clinical and radiographic evidence of early healing. Physiotherapy was supervised by a physical therapist and return to daily activities was guided by individual clinical progress at follow-up visits.

### 2.2. Outcome Assessment and Statistical Analysis

Constant, University of California at Los Angeles (UCLA), and Disabilities of the Arm, Shoulder and Hand (DASH) scores were administered face-to-face to all patients by orthopaedic surgeons who were not involved in the index surgical procedures. Data were analyzed using SPSS software (IBM SPSS Statistics version 22.0, Armonk, NY, USA). Patients were divided into subgroups based on the nature of their concomitant injuries and compared for functional outcomes. Data normality was assessed using the Shapiro–Wilk test. Independent samples *t*-tests and Mann–Whitney U tests were used for group comparisons. To assess the independent effect of thoracic trauma on functional outcomes, multivariate linear regression analysis was performed with DASH score as the dependent variable and thoracic trauma, age, sex, Ideberg fracture type, and concomitant ipsilateral upper extremity fracture as covariates, with the isolated glenoid fracture group as the reference category. Statistical significance was set at *p* < 0.05. Three patients who sustained both concomitant thoracic trauma and ipsilateral upper extremity fractures were included in the overall descriptive analysis (*n* = 31) and were counted within the respective groups for descriptive purposes; however, they were excluded from pairwise comparative group analysis due to the small subgroup size, which precluded independent statistical evaluation. A sensitivity analysis confirmed that these patients demonstrated favorable functional outcomes (mean Constant: 89.3, UCLA: 33.7, DASH: 0.3), comparable to the isolated fracture group, and their exclusion did not materially alter the overall conclusions of the study.

## 3. Results

The mean union time was 9.4 ± 2.4 weeks. Fracture union was determined by the operating surgeon based on radiographic findings and the absence of pain at clinical examination. The mean Constant score of all patients was 80.4 ± 11.3 (range: 57–94), the mean UCLA score was 29.6 ± 6.1 (range: 7–35), and the mean DASH score was 14.7 ± 14.8 (range: 0–52.5).

The Constant score was 85.5 ± 5.9 (range: 80–92) in the isolated scapular fracture group, 82.6 ± 11.7 (range: 57–94) in the ipsilateral upper extremity fracture group, and 70.9 ± 7.5 (range: 62–81) in the thoracic trauma group. The Constant score was significantly lower in the thoracic trauma group compared to both the isolated scapular fracture group (*p* = 0.012) and the ipsilateral upper extremity fracture group (*p* = 0.042), as assessed by the Mann–Whitney U test. No significant difference was observed between the isolated scapular fracture and ipsilateral upper extremity fracture groups (*p* = 0.825) (Table 4 and Table 5).

The UCLA score was 32.5 ± 3.0 (range: 29–35) in the isolated fracture group, 31.1 ± 3.2 (range: 26–35) in the ipsilateral upper extremity fracture group, and 24.6 ± 7.9 (range: 7–30) in the thoracic trauma group. The thoracic trauma group had significantly lower UCLA scores compared to both other groups (*p* = 0.010 and *p* = 0.012, respectively), as assessed by the Mann–Whitney U test. No significant difference was found between the isolated and ipsilateral fracture groups (*p* = 0.503), (Table 4 and Table 5).

The DASH score was 7.9 ± 9.2 (range: 0–17.5) in the isolated fracture group, 9.5 ± 9.9 (range: 0–30) in the ipsilateral upper extremity fracture group, and 29.3 ± 14.2 (range: 18.3–52.5) in the thoracic trauma group. DASH scores were significantly higher in the thoracic trauma group compared to both other groups (*p* = 0.003 and *p* = 0.006, respectively), as assessed by the Mann–Whitney U test. No significant difference was observed between the isolated and ipsilateral fracture groups (*p* = 0.604) (Table 4 and Table 5).

Multivariate linear regression analysis confirmed that concomitant thoracic trauma was an independent predictor of worse DASH scores (β = 12.75, 95% CI: 2.00–23.50, *p* = 0.031, R^2^ = 0.344), while age, sex, Ideberg fracture type, and concomitant ipsilateral upper extremity fracture did not reach statistical significance. A trend toward lower Constant and UCLA scores was also observed in the thoracic trauma group; however, these did not reach statistical significance in the regression model, likely attributable to the limited sample size.

None of the 31 patients developed postoperative infection, nonunion, implant-related irritation, or glenohumeral arthritis at final follow-up.

## 4. Discussion

In this study, we analyzed the clinical outcomes of 31 patients who underwent internal fixation with mini-plates using the MIPDS approach for glenoid fossa fractures after at least 2 years of follow-up and demonstrated that satisfactory results could be achieved. To the best of our knowledge, this is the first study in the literature to systematically examine the relationship between concomitant thoracic trauma and functional outcomes in patients surgically treated for glenoid fractures.

Lim et al. reported the outcomes of 10 patients (Ideberg type Ib and II) treated with the MIPDS approach at a mean follow-up of 14.1 months, with a mean Constant score of 84.7 ± 11.7 [8]. In the present study, the mean follow-up was substantially longer (67.1 ± 24.7 months) and the mean Constant score was 80.4 ± 11.3, which is comparable. Notably, our cohort additionally included Ideberg type III, IV, and V fractures, thereby broadening the demonstrated applicability of this surgical approach beyond the simpler fracture types previously reported.

Michelitsch et al. retrospectively evaluated 26 patients who underwent open or minimally invasive surgery for scapular fractures, demonstrating that open reduction and internal fixation is safe and effective with excellent functional results and low complication rates [4]. Mannambeth et al. reported satisfactory outcomes in 12 patients with extra-articular scapular fractures treated through a direct lateral column approach [13]. The functional scores achieved in our glenoid fracture cohort were comparable to those reported for extra-articular fractures in these series, suggesting that anatomical reduction of intra-articular fragments may effectively mitigate the inherent disadvantage associated with articular involvement.

The most clinically significant finding of this study is that patients with concomitant thoracic trauma had significantly worse outcomes across all three functional scores, whereas patients with concomitant ipsilateral upper extremity fractures did not differ significantly from those with isolated scapular fractures. This finding suggests that it is specifically the presence of thoracic trauma—rather than any concomitant injury per se—that adversely impairs functional recovery following glenoid fracture surgery. This was further supported by multivariate linear regression analysis, which identified thoracic trauma as an independent predictor of higher DASH scores (β = 12.75, *p* = 0.031), indicating a clinically meaningful increase in disability in this patient group.

Several mechanisms may account for this observation. First, thoracic trauma may prolong the rehabilitation period by restricting respiratory function and chest wall mobility during the healing process. Second, disruption of scapulothoracic motion due to rib fractures or associated pulmonary pathology may prevent full recovery of normal scapulothoracic kinematics, which are essential for optimal shoulder function. Third, although patients with overt neurological damage were excluded from this study, those sustaining thoracic injuries may have been exposed to higher-energy trauma overall, potentially resulting in unrecognized innervation damage and associated muscle weakness that would not have been apparent on standard clinical examination. None of these hypotheses could be confirmed with the available data, as scapulothoracic motion imaging was not performed in this series. To the best of our knowledge, no published study has previously examined the relationship between concomitant thoracic trauma and upper extremity functional outcomes following scapular surgery. Our findings therefore highlight an important and previously unrecognized prognostic factor that warrants further prospective investigation.

Regarding implant selection, Lim et al. used partially threaded cannulated screws [8], Michelitsch et al. used 3.5 mm or 2.4/2.7 mm LCP plates [4], and Mannambeth et al. used 3.5 mm or 2.7 mm plates [13]. In our series, 2.7 mm locking plates—commonly used in foot and ankle surgery—and 3 mm headless compression screws were employed. The absence of nonunion or implant removal procedures in any patient demonstrates the adequacy of these implants for glenoid fixation. At present, no implant specifically designed for the posterior or posteroinferior anatomy of the glenoid has been introduced; therefore, implant selection continues to be guided by individual clinical and surgical experience. It should be noted that foot and ankle locking plates were not originally designed for the posterior glenoid anatomy and may present certain constraints regarding plate profile and screw angulation. Nevertheless, the absence of nonunion, implant failure, or implant removal in any patient in our series supports their clinical adequacy for this indication.

This study has several limitations. First, the sample size is relatively small, which is inherent to the rarity of surgically treated glenoid fossa fractures. Despite this, statistically significant differences were identified between subgroups, supporting the clinical relevance of the findings. Second, although functional outcome data were collected prospectively during follow-up visits, the retrospective design limits causal inference. Third, the absence of scapulothoracic motion imaging data prevents full mechanistic characterization of the observed functional differences. Finally, the small number of patients with both thoracic and ipsilateral upper extremity injuries (*n* = 3) precluded their independent statistical analysis, and these patients were therefore excluded from comparative group analysis. Furthermore, although outcome assessments were performed by surgeons uninvolved in the index procedures, formal blinding to patients’ injury group assignments was not feasible given the retrospective nature of the study, which represents a potential source of measurement bias. Additionally, the relatively small subgroup sizes (*n* = 8, 9, and 14) limit the statistical power of the analysis, particularly for the Constant and UCLA scores in the regression model, and the possibility of type II error in these non-significant findings cannot be excluded.

## 5. Conclusions

The MIPDS approach using mini-plates provides safe and effective surgical fixation for Ideberg type Ib, II, III, IV, and V glenoid fractures, yielding satisfactory functional outcomes at a minimum follow-up of 2 years. Concomitant thoracic trauma is a significant negative predictor of functional recovery, and the possibility of inferior functional outcomes in this patient group should be taken into consideration. These findings carry direct clinical implications for preoperative patient counseling in cases of glenoid fracture associated with thoracic injury.

## Figures and Tables

**Table 1:** Demographic characteristics of the patients.

Characteristic	Value
Patient number (*n*)	31
Age, years (mean ± SD; range)	46.3 ± 16.6 (19–75)
Sex, *n* (%)	Male: 24 (77.4%)/Female: 7 (22.6%)
Affected side, *n* (%)	Right: 18 (58.1%)/Left: 13 (41.9%)
Follow-up duration, months (mean ± SD; range)	67.1 ± 24.7 (25–105)

**Table 2:** Concomitant injuries of the patients.

Injury Type	Number of Patients and Details
Isolated glenoid fracture	8
Concomitant ipsilateral upper extremity fracture	9 total: clavicle (3), clavicle + distal radius (1), distal radius (2), humeral shaft (1), forearm shaft (1), olecranon (1)
Concomitant thoracic trauma	14 (multiple rib fractures, pneumothorax, hemopneumothorax, pulmonary contusion)
Other concomitant trauma (outside ipsilateral extremity)	5

**Table 3:** Distribution of scapular fractures according to the Ideberg classification.

Fracture Type (Ideberg Classification)	Number of Patients (%)
Type Ib	9 (29.0%)
Type II	10 (32.2%)
Type III	2 (6.5%)
Type IV	6 (19.4%)
Type V	4 (12.9%)

**Table 4:** Pairwise comparisons of functional outcome scores between groups. (* Statistically significant (*p* < 0.05). DASH: Disabilities of the Arm, Shoulder and Hand; UCLA: University of California at Los Angeles; UE: upper extremity. *p* values based on Mann–Whitney U tests).

Comparison	Constant Score (Mean ± SD)	*p*	UCLA Score (Mean ± SD)	*p*	DASH Score (Mean ± SD)	*p*
Isolated vs. Thoracic trauma	85.5 ± 5.9 vs. 70.9 ± 7.5	0.012 *	32.5 ± 3.0 vs. 24.6 ± 7.9	0.010 *	7.9 ± 9.2 vs. 29.3 ± 14.2	0.003 *
Ipsilateral UE fracture vs. Thoracic trauma	82.6 ± 11.7 vs. 70.9 ± 7.5	0.042 *	31.1 ± 3.2 vs. 24.6 ± 7.9	0.012 *	9.5 ± 9.9 vs. 29.3 ± 14.2	0.006 *
Isolated vs. Ipsilateral UE fracture	85.5 ± 5.9 vs. 82.6 ± 11.7	0.825	32.5 ± 3.0 vs. 31.1 ± 3.2	0.503	7.9 ± 9.2 vs. 9.5 ± 9.9	0.604

Bold values indicate statistically significant results (*p* < 0.05).

**Table 5:** Descriptive functional outcome scores by concomitant injury group.

Group	Constant Score (Mean ± SD; Range)	UCLA Score (Mean ± SD; Range)	DASH Score (Mean ± SD; Range)
Isolated scapular fracture (*n* = 8)	85.5 ± 5.9 (80–92)	32.5 ± 3.0 (29–35)	7.9 ± 9.2 (0–17.5)
Concomitant thoracic trauma (*n* = 14)	70.9 ± 7.5 (62–81)	24.6 ± 7.9 (7–30)	29.3 ± 14.2 (18.3–52.5)
Concomitant ipsilateral UE fracture (*n* = 9)	82.6 ± 11.7 (57–94)	31.1 ± 3.2 (26–35)	9.5 ± 9.9 (0–30)
**All patients (*n* = 31)**	**80.4 ± 11.3 (57–94)**	**29.6 ± 6.1 (7–35)**	**14.7 ± 14.8 (0–52.5)**

UE: upper extremity; DASH: Disabilities of the Arm, Shoulder and Hand; UCLA: University of California at Los Angeles.

## Data Availability

The data presented in this study are available upon reasonable request from the corresponding author.
